# Incidence of atrial fibrillation in patients with atrioventricular nodal re-entrant tachycardia and its association with long-term outcome

**DOI:** 10.1016/j.hroo.2024.07.005

**Published:** 2024-07-14

**Authors:** Gesa von Olshausen, Nikola Drca, Astrid Paul-Nordin, Tara Bourke, Hamid Bastani, Serkan Saygi, Emma Svennberg, Finn Åkerström, Ott Saluveer, Mats Jensen-Urstad, Frieder Braunschweig

**Affiliations:** ∗ Department of Cardiology, Karolinska University Hospital, Stockholm, Sweden; † Medical Department I (Cardiology, Angiology, Pneumology), Klinikum rechts der Isar, Technical University of Munich, Munich, Germany; ‡ Heart and Lung Disease Unit, Department of Medicine, Huddinge, Karolinska Institutet, Stockholm, Sweden

**Keywords:** Atrioventricular nodal re-entrant tachycardia, Catheter ablation, Atrial fibrillation, Recurrence, Catheter ablation registry

## Abstract

**Background:**

Atrioventricular nodal re-entrant tachycardia (AVNRT) is the most common paroxysmal supraventricular tachycardia. We sought to investigate the incidence of atrial fibrillation in patients with electrophysiologically confirmed/ablated AVNRT and its association with transient ischemic attack (TIA)/stroke as well as mortality during long-term follow-up.

**Methods:**

From the Karolinska Ablation Registry, 2855 consecutive patients with a first-time ablation for AVNRT between 2005 and 2018 were analyzed.

**Results:**

Patients were 52.1 ± 15.9 years old and 59.3% were women. During follow-up of up to 10 years (median 6.0 years; interquartile range 3.3 to 9.2 years), new onset or recurrence of atrial fibrillation occurred in 317 (11.1%) patients (incidence rate 19 cases per 1000 person-years). Excluding those with history of atrial fibrillation, new onset of atrial fibrillation occurred in 153 (6.1%) patients. In multivariable analysis, history of atrial fibrillation, arterial hypertension, history of TIA/stroke, and heart failure remained independently associated with new onset or recurrence of atrial fibrillation during follow-up. Death of any cause and TIA/stroke occurred in 141 (4.9%) patients and 107 (3.7%) patients, respectively. In multivariable analysis, occurrence of atrial fibrillation during follow-up remained independently associated with both outcomes. The prevalence of atrial fibrillation according to age at the end of follow-up was high among young patients (<60 years of age: 12.7%; 60–69 years of age: 10.6%).

**Conclusion:**

In this large cohort of patients with diagnosed AVNRT, the incidence of atrial fibrillation was high (11.1%) during long-term follow-up. Occurrence of atrial fibrillation during follow-up remained independently associated with death for any cause as well as with TIA/stroke. Therefore, a closer monitoring for atrial fibrillation in patients with AVNRT including those at young age is advisable.

**Key Findings**

▪ In this large cohort of patients with electrophysiologically confirmed and ablated atrioventricular nodal re-entrant tachycardia, the incidence of atrial fibrillation was high, with 11.1% during 10 years of follow-up (incidence rate: 19 cases per 1000 person-years).
▪ The prevalence of atrial fibrillation according to age at the end of follow-up was high among young patients (<60 years of age: 12.7%; 60–69 years of age: 10.6%).
▪ Occurrence of atrial fibrillation during follow-up remained independently associated with death for any cause as well as with transient ischemic attack/stroke.

## Introduction

Atrioventricular nodal re-entrant tachycardia (AVNRT) is the most common type of paroxysmal supraventricular tachycardia encountered in adults^1^^,^^2^ and is more likely to begin at younger age.^3^ Catheter ablation has been shown to be superior to antiarrhythmic drugs and is usually offered as an option for first-line therapy for AVNRT.^2^^,^^4^ Atrial fibrillation is the most common sustained arrhythmia, affecting over 3% of the adult population, and an association between AVNRT and atrial fibrillation has been reported in previous studies.^5^, ^6^, ^7^, ^8^ As atrial fibrillation is associated with an increased risk of all-cause mortality and morbidity, including stroke,^9^ identification of patients at risk for atrial fibrillation is important to enable early diagnosis and management. However, there are little data from systematic analyses assessing the incidence of atrial fibrillation in patients with AVNRT in a large patient cohort with long-term follow-up. The purpose of this study was to investigate the incidence and risk factors for atrial fibrillation in patients with electrophysiologically confirmed and ablated AVNRT. In addition, the association of atrial fibrillation during follow-up with all-cause mortality and transient ischemic attack (TIA)/stroke was investigated.

## Methods

### Study protocol and setting

All patients who underwent electrophysiology procedures at the Karolinska University Hospital between January 2005 and September 2018 were analyzed. Relevant patient characteristics and procedural details were prospectively collected at the time of the ablation procedure and recorded in a computerized database.

The National Patient Registry and the Cause of Death Registry, administered by the Swedish Board of Health and Welfare, provided information on date and cause of death, further baseline comorbidities, and the cause-specific hospitalization as outcome defined according to the International Classification of Diseases–Tenth Revision codes. The personal identification number that all permanent Swedish citizens own served as unique identifier for each patient allowing merging of different registries.

Establishment of this analysis with linking of the previous registries was approved by the Swedish Ethical Review Authority (Etikprövningsmyndigheten) and conducted in accordance with the Declaration of Helsinki. According to the approval for this study, individual patient consent was not required.

### Study population and postablation follow-up

To identify patients with a reliable AVNRT diagnosis a cohort of patients with electrophysiologically confirmed and ablated AVNRT was selected. Consecutive patients (≥18 years of age at the time of index procedure) undergoing first-time catheter ablation of AVNRT between January 1, 2005, and September 301, 2018, were enrolled. History of atrial fibrillation at baseline was defined as atrial fibrillation within 6 years before index date. Catheter ablations of AVNRT were performed according to conventional and local standards as described previously.^10^ All patients were followed-up from the time of their index procedure until the date of death, emigration, or the end of the study (December 31, 2018).

### Study outcomes

Study outcomes were defined as new onset or recurrence of atrial fibrillation, death from any cause, and TIA/stroke. Atrial fibrillation and TIA/stroke were diagnosed during either in- or outpatient visits. Supplemental Table 1 displays the definition of the variables used in the current study.

### Statistical analysis

All continuous variables are presented as mean ± SD or median (interquartile range) and were compared by using Student’s *t* test. Categorical variables are expressed as frequency and percentage and were compared by chi-square tests. Clinical outcomes were examined within 10 years, which was deemed a meaningful period to reflect long-term follow-up. Univariate and multivariable backward logistic regression were performed to identify factors associated with new onset or recurrence of atrial fibrillation, death of any cause, and hospitalization for TIA/stroke. The multivariable model considered factors associated with a *P* value <.05 in univariate analysis. As all available AVNRT patients were included, no sample size calculation was performed. All statistical tests and confidence intervals were 2-sided, with a significance level of .05. Statistical analyses were performed using SPSS software, version 27 (IBM).

## Results

### Baseline and index procedure related characteristics of patients with first-time catheter ablation for AVNRT

Between January 1, 2005, and September 30, 2018, a total of 16,417 invasive electrophysiological procedures in 12,247 patients were performed. From this patient cohort, 2855 unique patients ablated for AVNRT fulfilled the inclusion criteria and were included in the analysis (Supplemental Figure 1). Most of these patients had a documented arrhythmia preablation (electrocardiogram- or smart device–documented arrhythmia 90.6% [n = 2587]; not documented arrhythmia 9.4% [n = 268]). Their mean age was 52.1 ± 15.9 years and 1692 (59.3%) patients were women. A total of 356 (12.5%) patients had a previous diagnosis of atrial fibrillation at baseline. All baseline clinical characteristics of the study participants are provided in Table 1 and all index procedure–related characteristics are presented in Table 2.

**Table 1:** Baseline characteristics of patients with catheter ablation for AVNRT

	AVNRT patients (n = 2855)	AVNRT patients without new onset/recurrence of AF during follow-up (n = 2538)	AVNRT patients with new onset/recurrence of AF during follow-up (n = 317)	*P* value
Age, y	52.1 ± 15.9	52.1 ± 15.8	52.1 ± 16.1	.993
Women	1692 (59.3)	1505 (59.3)	187 (59.0)	.481
BMI, kg/m^2,^∗	26.6 ± 12.2	26.5 ± 10.7	28.1 ± 21.8	.081
Ischemic heart disease	137 (4.8)	118 (4.6)	19 (6.0)	.328
Heart failure	49 (1.7)	29 (1.1)	20 (6.3)	<.001
Arterial hypertension	656 (23.0)	531 (20.9)	125 (39.4)	<.001
Diabetes mellitus	161 (5.6)	131 (5.2)	30 (9.5)	.002
Hyperlipidemia	178 (6.2)	150 (5.9)	28 (8.8)	.048
History of TIA/stroke	51 (1.8)	38 (1.5)	13 (4.1)	.003
CHA_2_DS_2_-VASc score	1.28 ± 1.02	1.24 ± 0.99	1.61 ± 1.17	<.001
History of AF	356 (12.5)	192 (7.6)	164 (51.7)	<.001

Values are mean ± SD or n (%).

AF = atrial fibrillation; AVNRT = atrioventricular nodal re-entrant tachycardia; BMI = body mass-index; CHA_2_DS_2_-VASc = congestive heart failure, hypertension, age ≥75 years, diabetes mellitus, prior stroke or transient ischemic attack or thromboembolism, vascular disease, age 65–74 years, sex category; TIA = transient ischemic attack.

∗ Data available in 69.4% of all patients.

**Table 2:** Index procedure–related characteristics in patients with catheter ablation for AVNRT

	AVNRT patients (n = 2855)
Energy delivery	
RF energy	229 (8.0)
Cryoenergy	2626 (92.0)
Fluoroscopy time, min	11.5 ± 9.2
Procedure time, min	114.2 ± 44.6
Periprocedural complications	
Artery puncture	93 (3.3)
Transient AV block∗	27 (0.9)

Values are n (%) or mean ± SD.

AV = atrioventricular; AVNRT = atrioventricular nodal re-entrant tachycardia; RF = radiofrequency.

∗ Transient AV block including AV block type II and III.

The mean duration of follow-up was 6.4 ± 3.7 years (median 6.0 years [interquartile range 3.3–9.2 years]).

### Outcome: atrial fibrillation, death of any cause, and TIA/stroke

**Figure 1:**
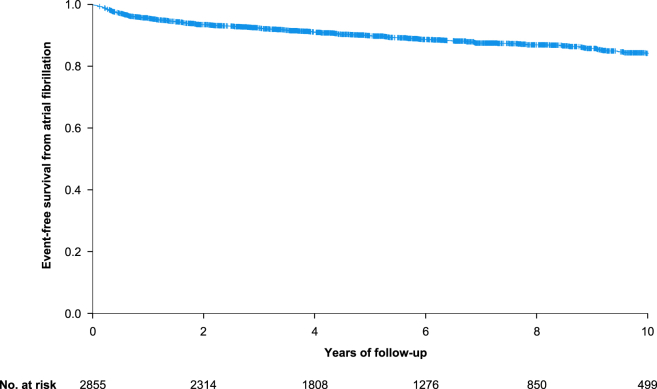
Kaplan–Meier analysis of event-free survival from atrial fibrillation after 10 year follow-up.

After a follow-up of up to 10 years, new onset or recurrence of atrial fibrillation occurred in 317 (11.1%) patients. The corresponding Kaplan-Meier-curve is provided in Figure 1. The incidence rate of atrial fibrillation was 19 cases per 1000 person-years. The mean period from AVNRT ablation to diagnosis of new onset or recurrence of atrial fibrillation was 2.6 ± 2.7 years. Excluding those with history of atrial fibrillation, new onset of atrial fibrillation occurred in 153 (6.1%) patients. When only analyzing patients with a history of atrial fibrillation already at baseline, recurrence of atrial fibrillation occurred in 164 (46.1%) patients. In multivariable analysis, history of atrial fibrillation, arterial hypertension, history of TIA/stroke, and heart failure remained independently associated with new onset or recurrence of atrial fibrillation in the total patient cohort (Supplemental Table 2). Excluding those with history of atrial fibrillation, history of TIA/stroke, arterial hypertension, and heart failure remained independently associated with new onset of atrial fibrillation in multivariable analysis (Supplemental Table 3). The prevalence of atrial fibrillation according to age at the end of follow-up is provided in Table 3.

**Table 3:** Prevalence of atrial fibrillation according to age at the end of follow-up

Age	All AVNRT patients (n = 2855) (%)
<60 y (n = 1491)	12.7
18–29 y (n = 177)	13.6
30–39 y (n = 249)	16.1
40–49 y (n = 467)	13.1
50–59 y (n = 598)	10.7
60–69 y (n = 679)	10.6
70–79 y (n = 490)	8.4
≥80 y (n = 195)	7.7
All ages	11.1

AVNRT = atrioventricular nodal re-entrant tachycardia.

Death of any cause occurred in 141 (4.9%) patients (for the corresponding Kaplan-Meier-curve, see Supplemental Figure 2) and cardiovascular death in 31 (1.1%) after a follow-up of 10 years. In multivariable analysis, heart failure, occurrence of atrial fibrillation during follow-up, arterial hypertension, and ischemic heart disease remained independently associated with death of any cause (Supplemental Table 4).

TIA/stroke occurred in 107 (3.7%) patients (for the corresponding Kaplan-Meier-curve, see Supplemental Figure 3) after a follow-up of 10 years. In multivariable analysis, history of TIA/stroke, arterial hypertension, and occurrence of atrial fibrillation during follow-up remained independently associated with TIA/stroke (Supplemental Table 5).

## Discussion

In this large cohort of patients with AVNRT, the incidence of atrial fibrillation was 11.1% during long-term follow-up. After adjustment for covariates, occurrence of atrial fibrillation during follow-up remained independently associated with death of any cause as well as with TIA/stroke. The prevalence of atrial fibrillation according to age at the end of follow-up was high among young patients.

To the best of our knowledge, this is the largest cohort of patients with AVNRT analyzed regarding the incidence of atrial fibrillation and its association with long-term outcome. In this study, the mean age with 52.1 ± 15.9 years at the time of AVNRT ablation was in the range of previous studies.^6^^,^^7^ The incidence of new onset or recurrence of atrial fibrillation during follow-up was high with 11.1% compared with general population-based data,^11^ which is in line with previous AVNRT studies^5^^,^^7^ or slightly higher.^6^ History of atrial fibrillation, arterial hypertension, history of TIA/stroke, and heart failure were independently associated with a higher risk to develop atrial fibrillation during follow-up, which is in accordance with previous studies.^5^^,^^7^ Noteworthy, in the current study the prevalence of atrial fibrillation according to age at the end of follow-up was high among young patients (<60 years of age: 12.7%; 60–69 years of age: 10.6%) compared with general population based data of a previous study (<60 years of age: 0.6%; 60–69 years of age: 4.2%).^12^

The high incidence of atrial fibrillation associated with AVNRT patients may be explained through different pathophysiologic pathways. One common and important factor associated with both arrhythmias is atrial fibrosis.^13^^,^^14^ In addition, most common sites of atrial ectopy that trigger atrial fibrillation are in or around the pulmonary veins,^15^ but rapid atrial activity (multiple wavelets, focal sources, and re-entrant mechanisms) from other sites (eg, coronary sinus and atrioventricular nodal tissue) can also initiate atrial fibrillation via the mechanism of re-entrant–mother rotor, as can AVNRT.^13^ Therefore, it has been shown that catheter ablation for AVNRT in patients without structural abnormalities can eliminate both AVNRT and atrial fibrillation.^16^ However, in patients with structural heart abnormalities, atrial fibrillation is likely to recure after AVNRT ablation. Hence, in patients undergoing AVNRT ablation, especially with structural heart disease and a history of atrial fibrillation, it may not be sufficient to treat AVNRT alone.

Occurrence of atrial fibrillation post–AVNRT ablation was relatively equally distributed during follow-up except from first year of follow-up in which around one-third of atrial fibrillation events occurred. It may be speculated that this higher event rate during first year of follow-up emerged due to increased attention to the palpitation symptoms after the procedure and a formerly incorrect classification of concomitant atrial fibrillation as (“only”) AVNRT.

Different from a recent analysis,^6^ the present study revealed that atrial fibrillation during follow-up remained independently associated with death of any cause. This is in accordance with former studies analyzing atrial fibrillation and increased mortality.^9^^,^^17^^,^^18^ Compared with Ozcan and colleagues,^6^ in the current study the sample size was larger and the incidence of atrial fibrillation was lower, which might be a possible explanation for this difference.

Occurrence of atrial fibrillation during follow-up remained independently associated with TIA/stroke. This is coherent with the literature because it is known that atrial fibrillation increases the risk of stroke 5-fold, although not homogeneously, depending on the presence of specific stroke risk factors.^9^ Considering the associated increased risk for death for any cause as well as with TIA/stroke with the occurrence of atrial fibrillation during long-term follow-up, the necessity of a closer clinical follow-up monitoring in patients with AVNRT is given.

### Limitations

Strengths of this study include the large cohort of consecutive patients and the opportunity to study their long-term outcomes through national registries with no patient loss during follow-up. However, some important limitations should be considered. All data have been collected at a single electrophysiology unit, and results may differ from other centers.

Follow-up postablation for atrial fibrillation did not include intensive monitoring such as regular Holter electrocardiogram, transtelephonic monitoring, or implantable loop recorder and was only documented by presentation of the patient during in- or outpatient visits. Therefore, it is possible that patients with silent atrial fibrillation may have been missed. However, our cohort represents real-world data with a clinic practical aspect.

A potential misclassification between AVNRT and atrial fibrillation cannot be excluded (ie, that an AVNRT may have been [mis]classified as atrial fibrillation, especially before AVNRT ablation and when patients were followed up by noncardiologists).

Only patients with electrophysiologically confirmed and ablated AVNRT were analyzed. Hence, results may differ to patients with suspected but not ablated AVNRT because AVNRT ablation itself may influence the incidence of atrial fibrillation during follow-up.

Administration of all kinds of treatment for atrial fibrillation (medical as well as ablative treatments) has not been provided in this study.

## Conclusion

In this large cohort of patients with diagnosed AVNRT, the incidence of atrial fibrillation was high, with 11.1% during long-term follow-up. Occurrence of atrial fibrillation during follow-up remained independently associated with death for any cause as well as with TIA/stroke. The prevalence of atrial fibrillation according to age at the end of follow-up was high among young patients. Therefore, a closer monitoring of patients with AVNRT including those at young age is advisable.
