# Supplementary material for: Incidence of atrial fibrillation in patients with atrioventricular nodal re-entrant tachycardia and its association with long-term outcome
Source: Heart Rhythm O2. 2024 Jul 14;5(8):538–42. doi: 10.1016/j.hroo.2024.07.005 (PMC11385394; doi:10.1016/j.hroo.2024.07.005)
Supplement: Supplementary Figure 1 [file mmc1.pdf]

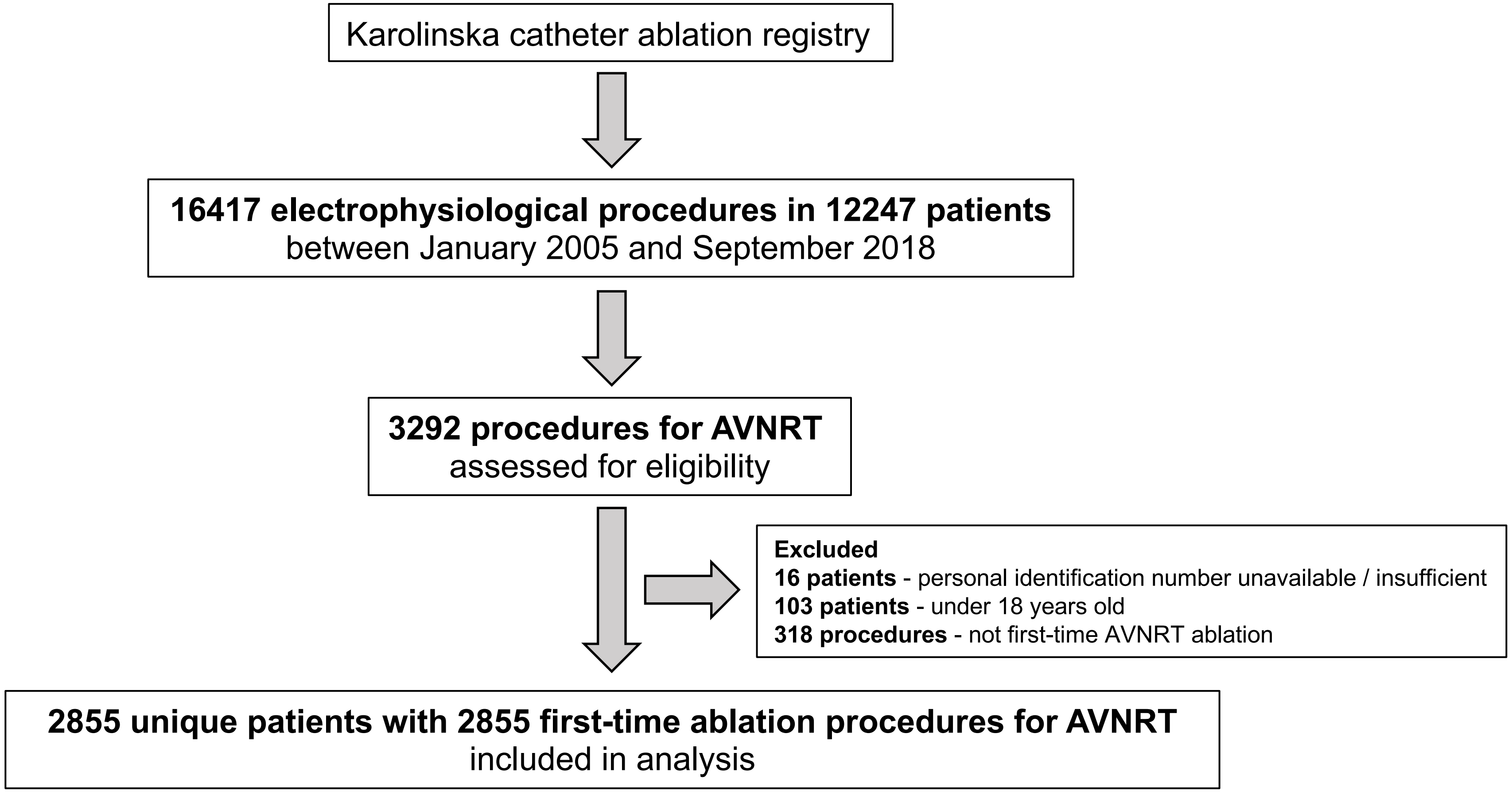

Figure Legend of Supplemental Figure 1:

PRISMA flow diagram showing the selection process for all patients
