# Supplementary material for: Incidence of atrial fibrillation in patients with atrioventricular nodal re-entrant tachycardia and its association with long-term outcome
Source: Heart Rhythm O2. 2024 Jul 14;5(8):538–42. doi: 10.1016/j.hroo.2024.07.005 (PMC11385394; doi:10.1016/j.hroo.2024.07.005)
Supplement: Supplementary Tables [file mmc4.docx]

Supplemental Table 1

Variable Definitions

| **Variable** | **Code** | **Registry** |
| --- | --- | --- |
| **Comorbidities** | | |
| **Heart failure** | ICD-10: I50 | NPR |
| **Arterial Hypertension** | ICD-10: I10-5 | NPR |
| **Diabetes** | ICD-10: E10-4 | NPR |
| **Hyperlipidemia** | ICD-10: E78 | NPR |
| **TIA /Stroke** | ICD-10: I60-4, I690-4, G45 | NPR |
| **Atrial fibrillation** | ICD-10: I48 | NPR |
| **Outcomes** | | |
| **Atrial fibrillation** | ICD-10: I48 | NPR |
| **TIA/Stroke** | ICD-10: I60-4, G45 | NPR |
| **All-cause death** | ICD-10: any | Cause of death |

ICD-10 codes were considered in any position for comorbidities and in primary (TIA/stroke) or primary/secondary position (atrial fibrillation) for outcomes

NPR = National Patient Registry, TIA = transient ischemic attack.

Supplemental Table 2

Logistic regression analysis for new-onset or recurrence of atrial fibrillation during 10 years of follow-up

| **Univariate analysis** | | |
| --- | --- | --- |
| **Variable** | **Odds ratio (95% CI)** | **p-value** |
| Age | 1.000 (0.998 – 1.007) | 0.993 |
| Sex, Female | 0.987 (0.779 – 1.252) | 0.916 |
| BMI | 1.006 (0.998 – 1.014) | 0.130 |
| Ischemic heart disease | 1.308 (0.794 – 2.154) | 0.292 |
| Heart failure | 5.826 (3.255 – 10.429) | <0.001 |
| Arterial hypertension | 2.461 (1.927 – 3.143) | <0.001 |
| Diabetes mellitus | 1.921 (1.268 – 2.909) | 0.002 |
| Hyperlipidemia | 1.542 (1.012 – 2.351) | 0.044 |
| History of TIA / stroke | 2.813 (1.482 – 5.340) | 0.002 |
| History of atrial fibrillation | 13.097 (10.049 – 17.070)  7 – 1.615) | <0.001 |
| **Multivariable analysis** | | |
| History of atrial fibrillation | 11.868 (9.058 – 15.549) | <0.001 |
| Arterial hypertension | 2.452 (1.226 – 4.906) | <0.001 |
| History of TIA / stroke | 2.050 (0.983 – 4.276) | 0.056 |
| Heart failure | 2.021 (1.534 – 2.664) | 0.011 |

CI = confidence interval, BMI = body-mass-index, TIA = transient ischemic attack

Supplemental Table 3

Logistic regression analysis for new-onset of atrial fibrillation during 10 years of follow-up (excluding those with history of atrial fibrillation)

| **Univariate analysis** | | |
| --- | --- | --- |
| **Variable** | **Odds ratio (95% CI)** | **p-value** |
| Age | 0.991 (0.981 – 1.001) | 0.093 |
| Sex, Female | 0.934 (0.667 – 1.307) | 0.690 |
| BMI | 1.005 (0.994 – 1.016) | 0.406 |
| Ischemic heart disease | 1.247 (0.619 – 2.510) | 0.537 |
| Heart failure | 3.740 (1.391 – 10.060) | 0.009 |
| Arterial hypertension | 2.956 (2.112 – 4.137) | <0.001 |
| Diabetes mellitus | 2.540 (1.502 – 4.297) | <0.001 |
| Hyperlipidemia | 1.416 (0.766 – 2.619) | 0.267 |
| History of TIA / stroke | 4.825 (2.248 – 10.355) | <0.001 |
| **Multivariable analysis** | | |
| History of TIA / stroke | 3.590 (1.640 – 7.860) | 0.001 |
| Arterial hypertension | 2.742 (1.948 – 3.859) | <0.001 |
| Heart failure | 2.682 (0.976 – 7.370) | 0.056 |

CI = confidence interval, BMI = body-mass-index, TIA = transient ischemic attack

Supplemental Table 4

Logistic regression analysis for death of any cause during 10 years of follow-up

| **Univariate analysis** | | |
| --- | --- | --- |
| **Variable** | **Odds ratio (95% CI)** | **p-value** |
| Age | 0.999 (0.998 – 1.010) | 0.827 |
| Sex, Female | 0.870 (0.618 – 1.223) | 0.423 |
| BMI | 0.962 (0.913 – 1.014) | 0.153 |
| Ischemic heart disease | 1.927 (1.038 – 3.575) | 0.038 |
| Heart failure | 10.399 (5.575 – 19.397) | <0.001 |
| Arterial hypertension | 4.172 (2.960 – 5.880) | <0.001 |
| Diabetes mellitus | 2.447 (1.434 – 4.173) | 0.001 |
| Hyperlipidemia | 2.336 (1.389 – 3.928) | 0.001 |
| History of TIA / stroke | 2.636 (1.105 – 6.287) | 0.029 |
| History of atrial fibrillation | 3.123 (2.131 – 4.575)  7 – 1.615) | <0.001 |
| Atrial fibrillation during follow-up | 5.215 (3.616 – 7.520) | <0.001 |
| **Multivariable analysis** | | |
| Heart failure | 5.715 (2.888 – 11.309) | <0.001 |
| Atrial fibrillation during follow-up | 3.752 (2.548 – 5.524) | <0.001 |
| Arterial hypertension | 3.334 (2.332 – 4.767) | <0.001 |
| Ischemic heart disease | 2.091 (1.087 – 4.023) | 0.027 |

CI = confidence interval, BMI = body-mass-index, TIA = transient ischemic attack

Supplemental Table 5

Logistic regression analysis for hospitalization for TIA/stroke during 10 years of follow-up

| **Univariate analysis** | | |
| --- | --- | --- |
| **Variable** | **Odds ratio (95% CI)** | **p-value** |
| Age | 0.999 (0.987 – 1.011) | 0.846 |
| Sex, Female | 0.945 (0.639 – 1.398) | 0.777 |
| BMI | 0.981 (0.932 – 1.033) | 0.466 |
| Ischemic heart disease | 0.764 (0.277 – 2.105) | 0.602 |
| Heart failure | 1.694 (0.518 – 5.537) | 0.383 |
| Arterial hypertension | 3.101 (2.099 – 4.581) | <0.001 |
| Diabetes mellitus | 1.568 (0.777 – 3.165) | 0.209 |
| Hyperlipidemia | 2.807 (1.612 – 4.887) | <0.001 |
| History of TIA / stroke | 5.917 (2.802 – 12.496) | <0.001 |
| History of atrial fibrillation | 1.871 (1.155 – 3.031)  7 – 1.615) | 0.011 |
| Atrial fibrillation during follow-up | 3.176 (2.038 – 4.948)  7 – 1.615) | <0.001 |
| **Multivariable analysis** | | |
| History of TIA / stroke | 4.104 (1.888 – 8.918) | <0.001 |
| Arterial hypertension | 2.601 (1.741 – 3.888) | <0.001 |
| Atrial fibrillation during follow-up | 2.503 (1.583 – 3.957) | <0.001 |

CI = confidence interval, BMI = body-mass-index, TIA = transient ischemic attack
